# Metabolomic Analysis Reveals Increased Aerobic Glycolysis and Amino Acid Deficit in a Cellular Model of Amyotrophic Lateral Sclerosis

**DOI:** 10.1007/s12035-015-9165-7

**Published:** 2015-05-12

**Authors:** Gabriel N. Valbuena, Milena Rizzardini, Sara Cimini, Alexandros P. Siskos, Caterina Bendotti, Lavinia Cantoni, Hector C. Keun

**Affiliations:** Department of Surgery and Cancer, Faculty of Medicine, Imperial College London, South Kensington, London SW7 2AZ UK; Department of Molecular Biochemistry and Pharmacology, IRCCS-Istituto di Ricerche Farmacologiche “Mario Negri”, 20156 Milan, Italy; Department of Neuroscience, IRCCS-Istituto di Ricerche Farmacologiche “Mario Negri”, 20156 Milan, Italy

**Keywords:** Amyotrophic lateral sclerosis, Amino acids, Glycolysis, Glutamine, Glutamate, Motor neuron, NSC-34, Serine, Cu/Zn superoxide dismutase, Pyruvate dehydrogenase kinase 1

## Abstract

Defects in energy metabolism are potential pathogenic mechanisms in amyotrophic lateral sclerosis (ALS), a rapidly fatal disease with no cure. The mechanisms through which this occurs remain elusive and their understanding may prove therapeutically useful. We used metabolomics and stable isotope tracers to examine metabolic changes in a well-characterized cell model of familial ALS, the motor neuronal NSC-34 line stably expressing human wild-type Cu/Zn superoxide dismutase (wtSOD1) or mutant G93A (G93ASOD1). Our findings indicate that wt and G93ASOD1 expression both enhanced glucose metabolism under serum deprivation. However, in wtSOD1 cells, this phenotype increased supply of amino acids for protein and glutathione synthesis, while in G93ASOD1 cells it was associated with death, aerobic glycolysis, and a broad dysregulation of amino acid homeostasis. Aerobic glycolysis was mainly due to induction of pyruvate dehydrogenase kinase 1. Our study thus provides novel insight into the role of deranged energy metabolism as a cause of poor adaptation to stress and a promoter of neural cell damage in the presence of mutant SOD1. Furthermore, the metabolic alterations we report may help explain why mitochondrial dysfunction and impairment of the endoplasmic reticulum stress response are frequently seen in ALS.

**Electronic supplementary material:**

The online version of this article (doi:10.1007/s12035-015-9165-7) contains supplementary material, which is available to authorized users.

## Introduction

Amyotrophic lateral sclerosis (ALS) is a progressive neurodegenerative disease affecting upper and lower motor neurons, usually leading to death in about 3–5 years after the onset of symptoms. Sporadic and familial forms of ALS (sALS and fALS) are clinically very similar and have no cure. fALS is caused by mutations in a heterogeneous group of genes [1]. Approximately 20 % of fALS and 5 % of apparent sALS patients have mutations in the Cu/Zn superoxide dismutase (*SOD1)* gene and the pathophysiological role of these mutations, which cause multiple changes in the different cell types of the central nervous system, are still not clear [1].

Rats, mice, and cells expressing mutant SOD1 proteins have been studied extensively as a model of ALS. SOD1 is primarily a cytosolic protein, but is also present in mitochondria, where it localizes mostly in the intermembrane space [2]. This enzyme catalyzes the dismutation of the superoxide radical, and therefore has a function in oxidative stress protection. SOD1 has also been shown to transmit signals from oxygen and glucose to regulate respiration [3].

Oxidative stress is clearly associated with disease onset in ALS and this seems to be just one aspect of a complex process leading to neurodegeneration [4, 1]. The mitochondria are the main source of reactive oxygen species (ROS), but they also provide the majority of metabolic energy through ATP formed by oxidative phosphorylation. Motor neuron mitochondria have been found to be morphologically and functionally altered in ALS patients and in mice and cells expressing mutant forms of SOD1 [4, 5]. These models showed bioenergetic defects [4, 6], and an early energy imbalance affecting survival was observed in the mutant G86RSOD1 transgenic mouse [7]. Abnormalities of energy metabolism are considered a potential factor contributing to the ALS disease phenotype as weight loss, hypermetabolism, and hyperlipidaemia have been observed in ALS patients [8]. In the central nervous system, energy homeostasis relies on metabolic interactions among different cell types, each with peculiar expression/regulation of energy metabolism enzymes and transporter proteins [9]. Glucose is the main neuronal energy source, but neurons also use other substrates, including lactate, the main end-product of aerobic glycolysis [10]. Interestingly, the failure of lactate exchange between oligodendrocytes and axons has been shown to contribute to motor neuron death in ALS patients and in G93ASOD1 mice [11].

Little information is available on whether or how mutant SOD1 in each cell type contributes to dysregulating energy metabolism, and its specific role in motor neuron death. Investigations into alterations to cellular metabolism associated with ALS may benefit from a comprehensive metabolomic approach. Metabolomic analysis has previously been applied to biofluids such as cerebrospinal fluid, where unique profiles were observed in patients carrying mutant *SOD1* [12].

In this study, we examined alterations to cellular metabolism in a previously characterized motor neuronal ALS model system, the murine neuroblastoma × spinal cord (NSC-34) cell line, stably expressing human wild-type (wt) SOD1 (wtSOD1) or mutant G93A (G93ASOD1) [13]. We employed a comprehensive metabolomic approach, involving untargeted profiling and stable isotope incorporation analysis using ^1^H nuclear magnetic resonance (^1^H NMR) spectroscopy and gas-chromatography-mass spectrometry (GC-MS). The untransfected and the SOD1-transfected NSC-34 cell lines were characterized under serum deprivation, which requires adaptation to oxidative and metabolic stress [14]. Previous work in this model showed that this stress was more toxic to motor neuronal cells expressing the G93ASOD1 protein compared to wtSOD1 [13]. Our results show that in response to serum deprivation, wtSOD1 ensured an increased supply of amino acids for protein and glutathione synthesis through enhanced glucose metabolism while this metabolic phenotype led to impairment of mitochondrial function and amino acid homeostasis when G93ASOD1 was expressed instead.

## Materials and Methods

### Materials

Flasks and plates were obtained from Corning Inc. High-glucose D-MEM and fetal bovine serum (FBS) were from Lonza, and high-glucose D-MEM without phenol red, geneticin (G418 sulfate), pyruvate, l-glutamine and penicillin/streptomycin were from GIBCO, Invitrogen. [U-^13^C_6_]glucose and [U-^13^C_5_]glutamine were from Cambridge Isotope Laboratories (Andover, MA, USA). Sodium dichloroacetate (DCA) was from Sigma-Aldrich.

The primary antibodies were hexokinase II, glyceraldehyde 3-phosphate dehydrogenase, pyruvate kinase isozyme M2, pyruvate dehydrogenase α subunit (Glycolysis Antibody Sampler Kit), lactate dehydrogenase A (#2012), all from Cell Signaling; pyruvate dehydrogenase kinase 1 (KAP-PK112) from Stressgen; hypoxia-inducible factor 1α from Novus Biologicals (NB100-479); peroxisome proliferator-activated receptor γ co-activator-1α from Santa Cruz Biotechnology (sc-13067); glutamine synthetase from Abcam (ab49873); monocarboxylate transporter 1 from Millipore (#AB1286-I); actin from Millipore (MAB 1501).

### Motor Neuronal ALS Model

NSC-34 is a hybrid cell line (mouse neuroblastoma N18TG2 × mouse embryonic spinal cord motor neurons) that expresses motor neuron features without the need for inducing agents in the medium [15] and is a well-characterized in vitro system for motor neuron biology. This cell line has been widely used to obtain models to investigate mechanisms of motor neuronal degeneration associated with mutant forms of human SOD1. This study investigated the effects of the glycine 93 to alanine (G93A) SOD1 point mutation.

Untransfected NSC-34 and the monoclonal NSC-34 lines stably expressing wtSOD1 (WT-NSC) and G93ASOD1 (G93A-NSC) used in this study [13] were grown in high-glucose DMEM supplemented with 5 % heat-inactivated FBS, 1 mM glutamine, 1 mM pyruvate, and antibiotics (100 IU/mL penicillin and 100 μg/mL streptomycin). The WT-NSC and G93A-NSC cell lines were kept in selection by adding 0.5 mg/mL geneticin. The lines were cultured simultaneously, subcultured in parallel every seven days and never maintained beyond passage 20 in order to limit any potential effects of senescence.

### Treatments and Isotopic Labeling

The standard treatment was culture without serum for 22 h. The cell lines, all at the same passage number, were seeded into multi-well plates at a density of 7630 cells/cm^2^ per well in the standard culture medium with/without geneticin and maintained at 37 °C in a humidified atmosphere with 5 % CO_2_ for 96 h. The medium was then removed and all the lines were exposed for 22 h to medium without geneticin, phenol red, and FBS. Medium and cells were then processed according to the protocol of each analysis.

For the experiments with DCA, the compound (2.5 or 5.0 mM for 22 h) was added to cells when the medium without FBS was added.

For isotopic labeling experiments, cells were cultured in six-well plates for 96 h in standard culture medium. The medium was then removed and cells were rinsed with 1X PBS. Cells were then cultured for 22 h in DMEM with no FBS containing either [U-^13^C_6_]glucose (25 mM) or [U-^13^C_5_]glutamine (1 mM). The media was collected and cells were rinsed with cold Ringer’s solution. Metabolites were extracted and metabolism was quenched by immediately adding cold methanol. The cell extracts were dried down using a centrifugal evaporator, and the media and cell extracts were stored at −70 °C until metabolomic analysis.

Replicate plates were prepared to determine the protein content, with the bicinchoninic acid (BCA) protein assay kit (Pierce, Rockford, IL, USA) for normalization.

### ^1^H NMR Spectroscopy of Cell Culture Media

Five hundred fifty microliters of culture medium and 50 μL of 11.6 mM sodium 3-trimethylsilyl-[2,2,3,3-^2^H_4_]-1-propionate (TSP) in deuterium oxide (D_2_O) as internal standard were mixed and transferred to glass 5-mm NMR tubes for analysis. High-resolution ^1^H NMR spectra of cell culture media were acquired at 14.1 T (600.13 MHz ^1^H frequency) using a Bruker AVANCE 600 spectrometer (BrukerBiospin, Rheinstetten, Germany). All spectra were acquired using a CPMG pulse sequence, and the sum of 64 free induction decays (FIDs) were collected into 64 k data-points with a spectral width of 12,019.230 and 0.3 Hz line broadening.

### GC-MS of Intracellular Metabolites

Aqueous metabolites were separated from the intracellular extract using a 2:1:3 chloroform/methanol/water extraction method. The aqueous portion of the extract was separated and lyophilized before analysis.

Samples for metabolic profiling were derivatised for GC-MS by a two-step methoximation/silylation derivatisation procedure. 2,3,3-d_3_-Leucine (20 μL, 1 mM) and [U-^13^C_6_]glucose (20 μL, 1 mM) were added to the samples as derivatization standards. The dried samples were first methoximated with a solution of 20 mg/mL methoxyamine hydrochloride (20 μL) in anhydrous pyridine at 30 °C for 90 min, then silylated with N-methyl-N-trimethylsilyltrifluoroacetamide (MSTFA) with 1 % trimethylchlorosilane (TMCS; 80 μL) at 37 °C for 30 min. After derivatization, 2-fluorobiphenyl in anhydrous pyridine (10 μL, 1 mM) was added to the samples as an injection standard.

Samples for stable isotope incorporation analysis were derivatised for GC-MS with a similar two-step methoximation/silylation procedure, but using 80 μL of *N*-*tert*-butyldimethylsilyl-*N*-methyltrifluoroacetamide with 1 % *tert*-butyldimethylchlorosilane at 70 °C for 60 min instead of MSTFA with 1 % TMCS.

GC-MS analysis was done on an Agilent 7890 gas chromatograph connected to an Agilent 5975 MSD (Agilent Technologies UK Ltd.). Samples were injected with an Agilent 7683 autosampler injector into deactivated splitless liners using the FiehnLib settings [16].

### Metabolomic Data Analysis

^1^H NMR spectra were imported and manipulated in Matlab (Mathworks, Natick, MA, USA) using in-house code for automatic phasing, baseline correction, and referencing chemical shifts to the TSP resonance at δ 0. The observed resonances were assigned to specific metabolites by database matching using the Chenomx NMR suite (Chenomx Inc., Alberta, Canada) and the Human Metabolome Database (HMDB).

GC–MS profiling data were processed by deconvolution with AMDIS using the Fiehn library, followed by an additional step as previously described [17] for visual inspection of peak quality (including match to retention time) and back-filling the sample matrix so that all metabolites had a numerical value for all samples, based on the quantitation ion for each metabolite, taken from the Fiehn library. Data were normalized to internal standards and sample protein content.

GC-MS stable isotope incorporation data were processed with an in-house MatLab package (developed by Dr. Gregory Tredwell) for the quantification of mass isotopomer distributions and correction for natural isotope abundances. Further processing and normalization was carried out on R.

### MTT Assay

Reduction of 3-(4,5-dimethylthiazol-2-yl)-2,5 diphenyltetrazolium bromide (MTT) to formazan crystals by cellular dehydrogenases was used as a measure of the number of living cells, as previously described [13].

### Caspase Activity

Caspase 3/7 activity was determined with a luminescence assay (Caspase-Glo 3/7 Assay, Promega) according to the manufacturer’s protocol.

### Lactate Release

Lactate release in the culture medium was determined with a spectrophotometric method adapted to analysis with a 96-well plate, as previously described [18].

### SDS-PAGE and Western Blot

The cell lines were grown and treated in 12- or 6-well plates as previously described. The treated plates were put on ice, the medium was removed and each well was washed twice with PBS 1X. Thirty to fifty microliters of RIPA buffer containing protease-(P8340, Sigma-Aldrich) and phosphatase-inhibitor (PhosSTOP, Roche Applied Science) cocktails were added to each well; the plates were then left on ice for 10 min. Cell lysates were collected, left on ice for 30 min, vortexed and centrifuged at 4 °C at 8000×*g* for 10 min. The supernatants were stored in aliquots at −80 °C until used to determine the expression levels of the proteins of interest. For nuclear extracts, cells were lysed in NE-PER extraction reagent (Pierce) according to the manufacturer’s protocol.

Proteins (30–50 μg) were separated by electrophoresis on polyacrylamide gels (7.5–12 %), then electroblotted on nitrocellulose membranes. The blots were exposed to the primary antibodies and to the appropriate HRP-conjugated secondary antibodies. Actin was used as a loading control. Protein bands were detected with the ECL detection system (Amersham Biosciences, Little Chalfont, UK). Films were scanned and band intensities were obtained with QuantityOne (Bio-Rad).

### Statistical Analysis

The statistical significance of the data was tested by one-way ANOVA followed by Tukey’s post hoc test. Metabolomic data were subjected to Benjamini and Hochberg False-Discovery Rate (FDR) correction for multiple testing. All data are presented as mean ± SEM unless otherwise stated. Statistical analyses were done with GraphPad Prism version 6.01 or in the R statistical environment.

## Results

### Serum Deprivation Causes Selective Cytotoxicity and Increased Lactate Production in NSC-34 Cells Expressing G93ASOD1

**Fig. 1:**
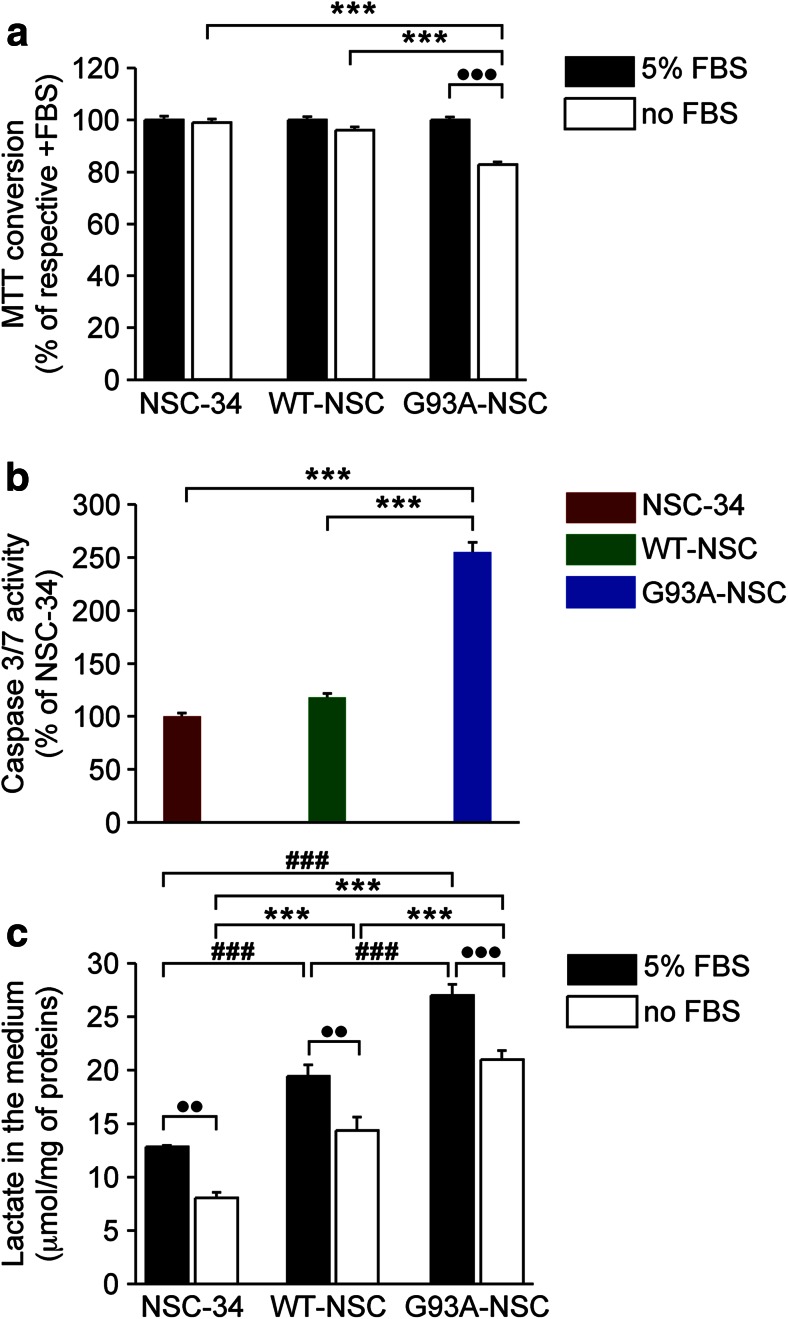
Serum deprivation causes selective cytotoxicity, caspase 3/7 activation, and increased lactate formation in G93ASOD1 cells. **a** The viability of the NSC-34, WT-NSC, and G93A-NSC cell lines was evaluated with the 3-(4,5-dimethylthiazol-2-yl)-2,5 diphenyltetrazolium bromide (MTT) assay after culture with or without 5 % fetal bovine serum (FBS) for 22 h. Percentages of the MTT conversion after culture with serum (100 %; *n* = 12) are shown. **b** Activation of caspase 3/7 of the NSC-34, WT-NSC, and G93A-NSC cell lines cultured without 5 % serum for 17 h. Values are percentages of the NSC-34 activity (100 %; *n* = 15). **c** Levels of lactate in the medium (μmol/mg of protein; *n* = 8) of the NSC-34, WT-NSC, and G93A-NSC cell lines cultured with or without 5 % serum for 22 h. All values are mean ± s.e.m. For each parameter, statistical significance of differences was assessed by one-way ANOVA and Tukey’s post hoc test comparing the levels of the different cell lines with or without 5 % serum (respectively ###*p* < 0.001 and ***p < 0.001) or the level in each cell line without 5 % serum versus respective with 5 % serum (●●*p* < 0.01; ●●●*p* < 0.001)

The WT-NSC and the G93A-NSC cell lines used in this study have been described previously [13]. Serum deprivation is a stress requiring adaptation to oxidative and metabolic stress. Culturing the cell lines without serum for 22 h led to a statistically significant loss of cell viability only in the G93A-NSC (18 % decrease compared to culture in medium with 5 % serum, Fig. 1a). This loss of viability was preceded by a 2.5-fold increase in caspase 3/7 activity compared to NSC-34 and WT-NSC (*p* < 0.001, Fig. 1b). The increased susceptibility of G93ASOD1 motor neuronal cells to serum deprivation [14, 13], as well as the caspase-inducing activity of mutant SOD1, has previously been reported. This pointed to mitochondrial involvement in the death of motor neurons, although death was driven by a caspase-independent pathway [19]. Previous work in the same cell model showed no difference in caspase activation among the three lines after just 4 h of serum deprivation [20], indicating that caspase activation occurred after longer exposure to serum deprivation, and not under culture with 5 % FBS.

We reported that high extracellular lactate levels are a phenotypic marker of altered metabolic response to stress in cells expressing a high or low level of G93ASOD1 [18, 20]. Initial assessments of glycolytic rates through spectrophotometric measurements of lactate in the medium showed significantly lower levels in culture without serum than with serum for all the cell lines, as expected in a condition that slows proliferation (Fig. 1c, *p* < 0.001). However, lactate was significantly higher in the medium of the WT-NSC and G93A-NSC than the NSC-34 both with serum (+51 and +110 %, respectively) and without (+79 and +161 %). In addition, lactate levels in the G93A-NSC line were significantly higher than that of the WT-NSC (+39 % with serum and +46 % without, Fig. 1c). These results suggest increased glycolysis in the SOD1-transfected lines compared to the untransfected NSC-34, with the effect being more marked in G93A-NSC than WT-NSC, and in culture without serum. As serum deprivation revealed the differences in toxicity of the mutant SOD1, we further characterized the metabolic phenotype of the cells in this culture condition.

### Metabolic Profiling Reveals Increased Levels of Glycolysis and Glutaminolysis in the G93A-NSC Line

**Fig. 2:**
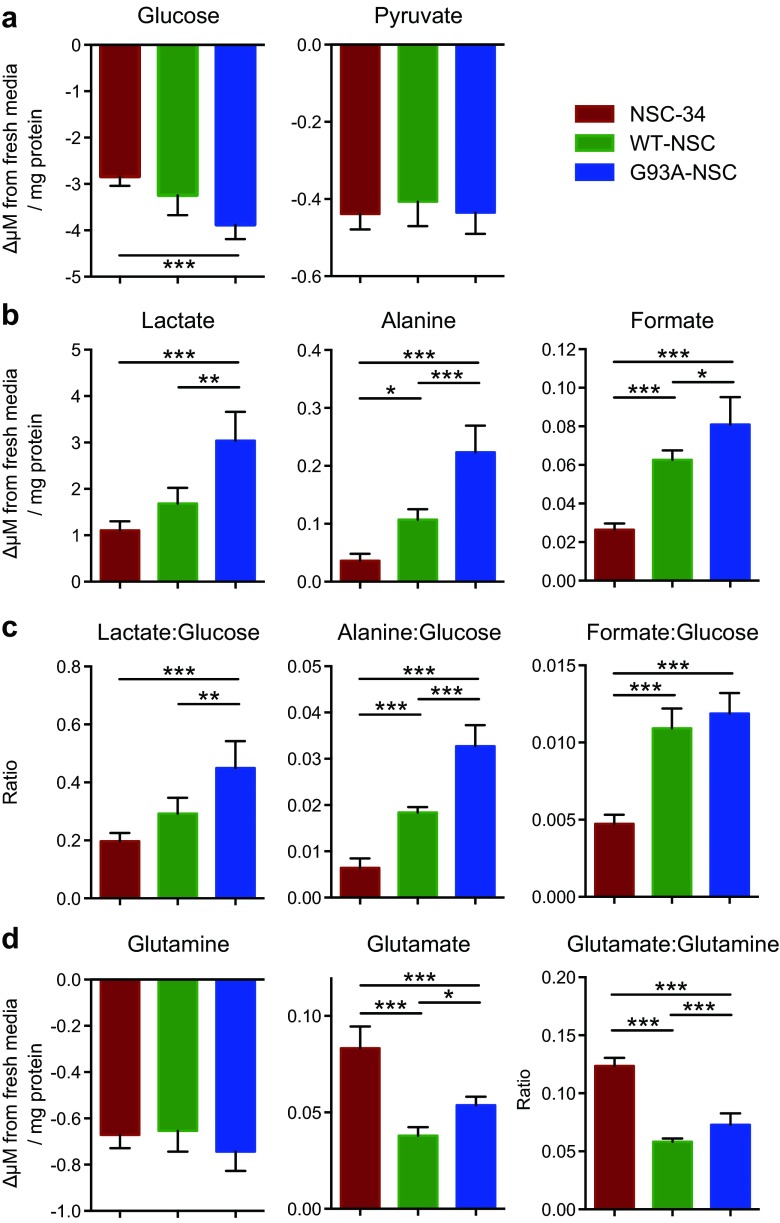
Extracellular metabolome characterization reveals regulation of glycolysis and glutaminolysis with expression of wt- and G93ASOD1. The NSC-34, WT-NSC, and G93A-NSC cell lines were cultured for 22 h without serum, the media was collected and metabolites were determined by ^1^H NMR spectroscopy. Negative values indicate consumption from the media and positive values indicate net release. Lactate, alanine, formate, and glutamate are not present in fresh culture medium. Changes in concentrations of **a** glucose and pyruvate, **b** lactate, alanine, and formate compared to fresh medium. **c** Ratios of lactate, alanine, and formate produced per unit of glucose consumed. **d** Glutamine consumption, glutamate production, and ratio of glutamate produced per unit of glutamine consumed. All values are mean ± s.e.m. (*n* = 4,*p < 0.05, **p < 0.01, ***p < 0.001 after one-way ANOVA with False-Discovery Rate (FDR) correction for multiple testing and Tukey’s post hoc test)

We first profiled the extracellular metabolome after serum deprivation for 22 h through ^1^H NMR spectroscopy of the cell media. The consumption of glucose in the G93A-NSC line was 37 % higher than in the NSC-34 (*p* < 0.001), with WT-NSC showing intermediate levels (Fig. 2a). We confirmed the progressively higher lactate production in G93A-NSC compared to WT-NSC and in WT-NSC compared to NSC-34 (Fig. 2b). In parallel, alanine and formate levels were both higher in the media from the G93A-NSC line (+109 and +29 %, respectively) than from WT-NSC, and in the media from WT-NSC (+197 and +137 %) than from NSC-34. The same trend was observed in these metabolites in media of the G93A-NSC after culture in 5 % FBS (Supplementary Fig. 1), indicating that these changes were not solely due to caspase activation. Normalizing metabolite release to glucose consumption, which gives a molar ratio independent of cell number, confirmed the significant increases in extracellular lactate and alanine in G93A-NSC compared to WT-NSC (+54 and +78 %, respectively; Fig. 2c). Since both metabolites can be derived directly from pyruvate, this suggested a different fate of glucose-derived pyruvate in the lines expressing wt- or G93ASOD1. Overall, these results indicated that while wtSOD1 promoted glycolysis, G93ASOD1 may further increase lactate and alanine formation from glucose. The significant differences in formate release into the medium across all three lines, as well as the significant increase in the ratio of formate release to glucose uptake is of interest, as formate plays a key role in cellular one-carbon metabolism, although the biological impact of these changes will require further investigation.

Glutamate levels in the media of WT-NSC and G93A-NSC lines were lower (−54 and -35 %, respectively, *p* < 0.01) compared to NSC-34. However, glutamate levels were higher in G93A-NSC than WT-NSC (+41 %, *p* < 0.05). There were no significant differences in the consumption of glutamine from the medium among the three lines, although the ratios of glutamate produced to glutamine consumed were significantly different (NSC-34>G93A-NSC>WT-NSC; Fig. 2d). These results suggest that SOD1 might regulate glutamate metabolism, and that mutant G93ASOD1 increased the extracellular accumulation of glutamate compared to wtSOD1.

**Fig. 3:**
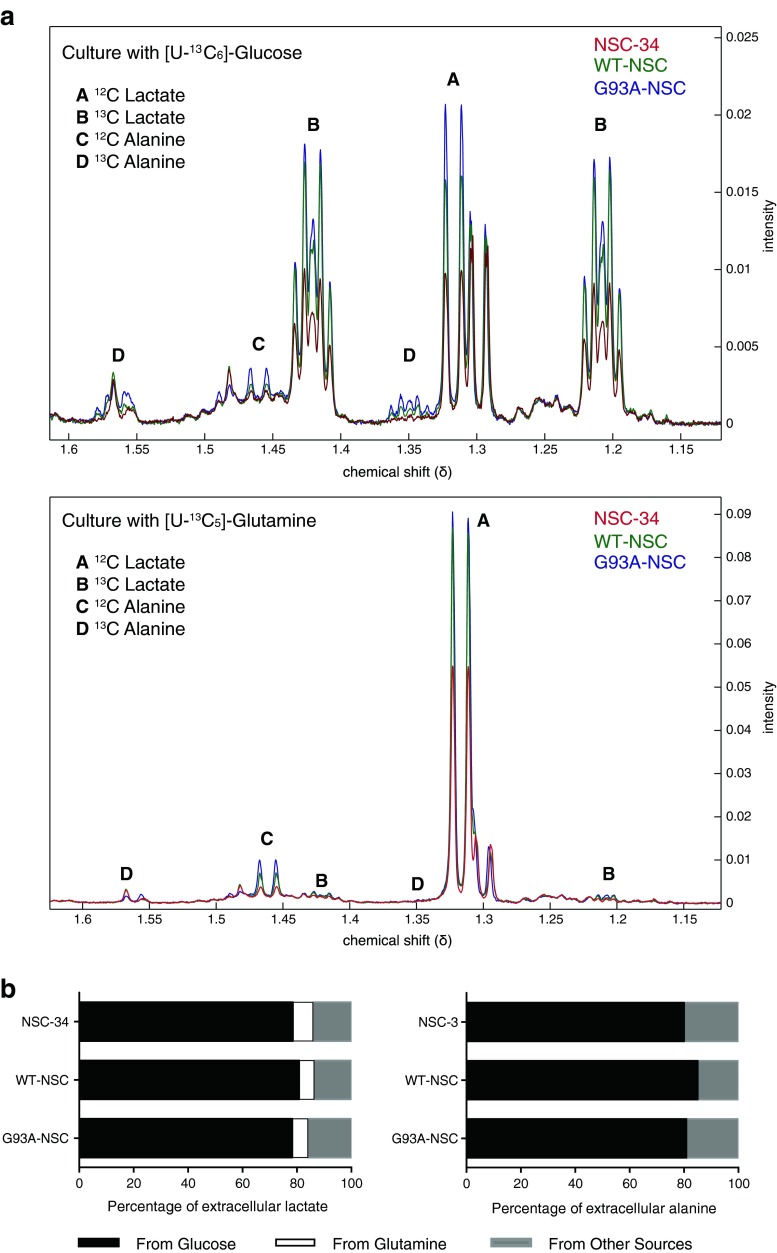
Glucose is the main source of lactate and alanine production in NSC-34 cells, both untransfected and SOD1-transfected. **a** The NSC-34, WT-NSC, and G93A-NSC cell lines were cultured for 22 h without serum and with [U-^13^C_6_]glucose (upper panel) or [U-^13^C_5_]glutamine(lower panel). Figure shows median ^1^H NMR spectra. **b** Mean calculated percentages of lactate and alanine derived from glucose or glutamine based on the ^1^H NMR data (*n* = 5)

To confirm the source of extracellular lactate and alanine, we cultured the cell lines in [U-^13^C_6_]glucose or [U-^13^C_5_]glutamine under serum deprivation and characterized the culture media by ^1^H NMR spectroscopy (Fig. 3a). The vast majority of lactate and alanine (∼80 %) came from glucose in all lines (Fig. 3b) and there were only very minor differences in the fraction derived from either glucose or glutamine between WT-NSC and G93A-NSC (Fig. 3b).

**Fig. 4:**
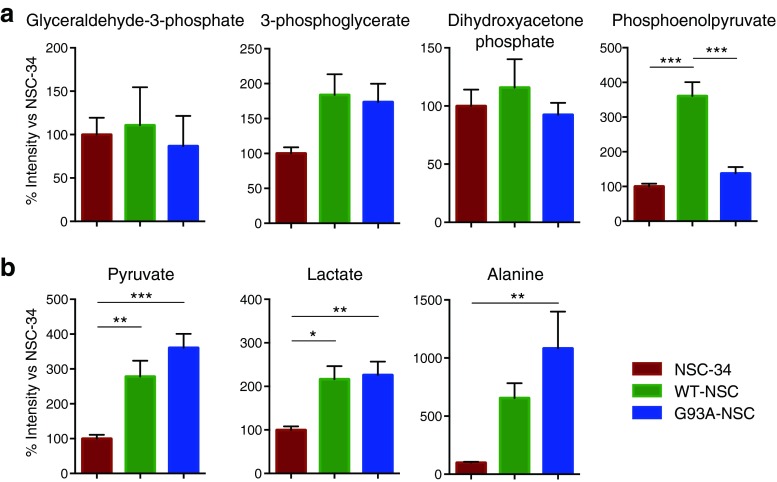
Intracellular levels of pyruvate and its metabolites lactate and alanine are high in cells expressing human SOD1. The NSC-34, WT-NSC, and G93A-NSC cell lines were cultured for 22 h without serum. Intracellular glycolytic metabolites were measured by GC-MS and expressed as percentages of the levels in the untransfected NSC-34 line. **a** Levels of the glycolytic intermediates glyceraldehyde-3-phosphate, 3-phosphoglycerate, dihydroxyacetone phosphate, and phosphoenolpyruvate. **b** Levels of the glycolytic products pyruvate, lactate, and alanine. All values are mean ± s.e.m. (*n* = 6,*p < 0.05, **p < 0.01, ***p < 0.001 after one-way ANOVA with FDR correction for multiple testing and Tukey’s post hoc test)

We then probed the intracellular metabolite profile using GC-MS analysis of cellular extracts. There were no significant differences among the three cell lines in three out of four detectable glycolytic intermediates (glyceraldehyde-3-phosphate, 3-phosphoglycerate, and dihydroxyacetonephosphate, Fig. 4a) although the level of 3-phosphoglycerate, the glycolytic precursor of serine, was 70-80 % higher in WT-NSC and G93A-NSC than in NSC-34. However, levels of phosphoenolpyruvate (PEP) were strikingly higher only in the WT-NSC line (+261 and +162 % compared to NSC-34 and G93A-NSC, respectively, *p* < 0.001, Fig. 4a). Pyruvate, the glycolytic precursor of lactate, was significantly elevated in the WT-NSC and G93A-NSC lines compared to NSC-34 (+178, and +261 %, respectively, Fig. 4b). The glycolytic products lactate and alanine were also higher in the WT-NSC and G93A-NSC than the NSC-34 line (Fig. 4b). Consistent with results shown in Fig. 2, these findings indicate increased glycolytic flux in the transfected lines (which increased lactate and alanine production) and that the differences between wt- and G93ASOD1-expressing cells might be due to different regulation of pyruvate metabolism after the formation of PEP and not to a change in the upstream steps of glycolysis.

**Fig. 5:**
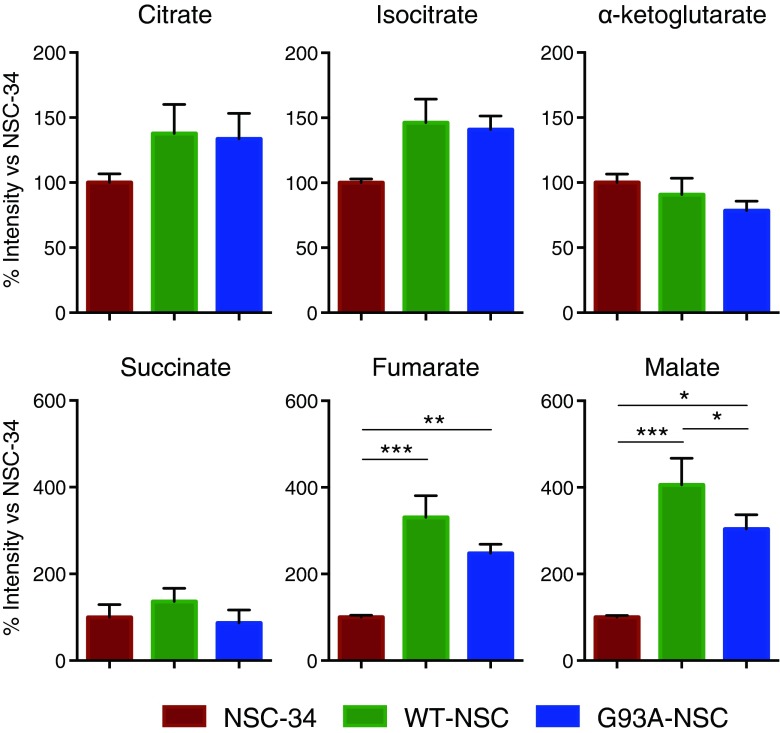
Metabolomic analysis of intracellular TCA cycle intermediates shows accumulation of fumarate and malate in wt- and G93ASOD1 cells. The NSC-34, WT-NSC, and G93A-NSC cell lines were cultured for 22 h without serum. Intracellular TCA cycle metabolites were measured by GC-MS and expressed as percentages of the levels in the untransfected NSC-34 line. All values are mean ± s.e.m. (*n* = 6,*p < 0.05, **p < 0.01, after one-way ANOVA with FDR correction for multiple testing and Tukey’s post hoc test)

There was a trend towards higher intracellular concentrations in four out of six detectable tricarboxylic acid (TCA) cycle intermediates for both WT-NSC and G93A-NSC compared to NSC-34, including citrate and isocitrate (+30–40 %, n.s.) and fumarate and malate (+145–305 %, statistically significant, Fig. 5). Elevated cytosolic fumarate can create a pseudohypoxic state, characterized by activation of hypoxia-inducible factor 1α (HIF-1α) with consequent increases of glycolysis and repression of respiration in normoxic conditions [21, 22]. The fumarate and malate levels were slightly lower in the G93A-NSC line than in WT-NSC (−25 and −39 %). TCA cycle enzymes are regulated through product inhibition, so accumulation of TCA cycle intermediates, particularly citrate, may cause a decrease in the metabolic activity of the TCA cycle with wt- and G93ASOD1 expression. This is consistent with diversion of pyruvate from the TCA cycle towards lactate and alanine synthesis.

**Fig. 6:**
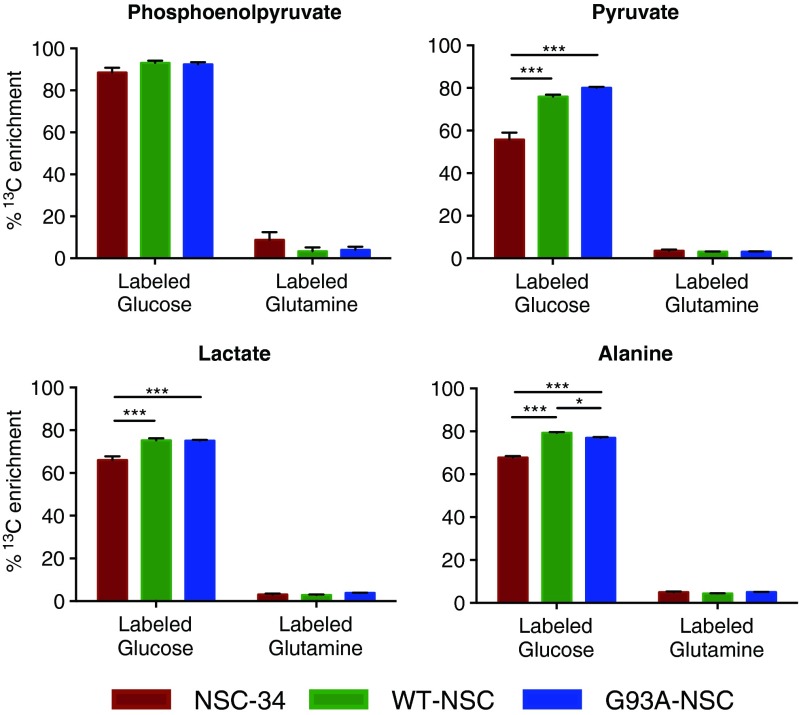
Wt- and G93ASOD1 cells show increased incorporation of glucose- but not glutamine-derived carbon into pyruvate, lactate, and alanine. The NSC-34, WT-NSC, and G93A-NSC cell lines were cultured for 22 h without serum and with [U-^13^C_6_]glucose or [U-^13^C_5_]glutamine. Histograms show the percentages of the intracellular pools of phosphoenolpyruvate, pyruvate, lactate, and alanine enriched with ^13^C after culture with labeled glucose or glutamine. All values are mean ± s.e.m. (*n* = 5, *p < 0.05, **p < 0.01, ***p < 0.001 after one-way ANOVA with FDR correction for multiple testing and Tukey’s post hoc test)

We then used GC-MS to characterize the incorporation of stable isotopes into intracellular metabolites from [U-^13^C_6_]glucose and [U-^13^C_5_]glutamine and confirmed that ∼60 % of pyruvate, lactate, and alanine carbons were glucose-derived in parental NSC-34 cells (Fig. 6). This fraction rose to ∼80 % in WT-NSC and G93A-NSC, with only alanine enrichment from glucose showing any (minor) difference between the G93A-NSC and the WT-NSC (77 and 79 %, respectively). Levels of PEP derived from glucose remained unchanged (∼90 %) irrespective of wt- or G93ASOD1 expression, and levels of glutamine-derived carbon were consistently low (<10 %) for all metabolites (Fig. 6).

**Fig. 7:**
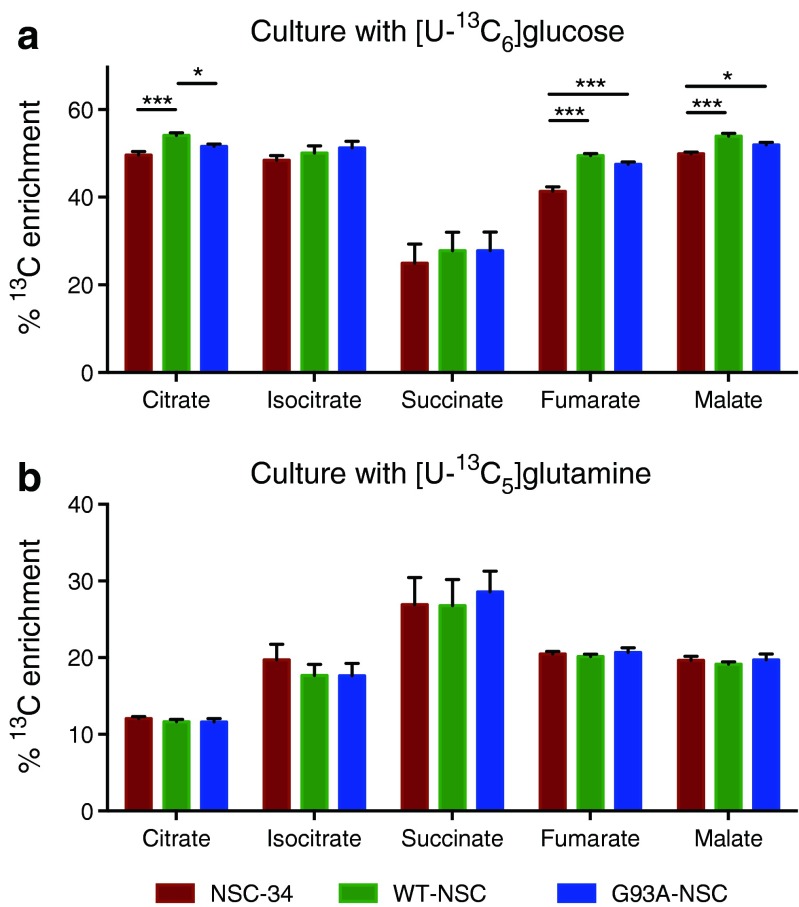
Incorporation of glucose- and glutamine-derived carbon into TCA cycle intermediates in wt- and G93ASOD1 cells. The NSC-34, WT-NSC, and G93A-NSC cell lines were cultured for 22 h without serum and with [U-^13^C_6_]glucose or [U-^13^C_5_]glutamine. Histograms show the percentages of the intracellular pools of TCA cycle intermediates enriched with ^13^C after culture with **a** labeled glucose or **b** labeled glutamine. All values are mean ± s.e.m. (*n* = 5, *p < 0.05, **p < 0.01, ***p < 0.001 after one-way ANOVA with FDR correction for multiple testing and Tukey’s post hoc test)

We next examined ^13^C enrichment in the intracellular pools of TCA cycle intermediates (Fig. 7). For citrate, isocitrate, fumarate, and malate, the contribution of carbon from glucose (∼40–55 %) was greater than for glutamine (10–20 %) across all cell lines, whereas the contribution (20–25 %) was approximately the same for succinate. The reduction in the fraction of succinate derived from glucose, as well as the corresponding increase in the fraction derived from glutamine when compared to isocitrate indicated that the main entry point of glutamine carbon into the TCA cycle was through α-ketoglutarate after initial conversion into glutamate. We saw significant increases in glucose-derived carbon in fumarate and malate for both WT-NSC and G93A-NSC (Fig. 7), complementing the larger pool size of these metabolites (Fig. 5), as well as a significant reduction of glucose-derived carbon in citrate in the G93A-NSC line compared to WT-NSC (Fig. 7). However, although these changes were significant, the differences were very small, and the biological impact of these changes will still require further investigation.

Closer examination of the mass isotopologue distributions for citrate (Supplementary Fig. 2a) in the presence of [U-^13^C_6_]glucose indicated that this effect was primarily driven by a reduction of the M+5 isotopologue fraction with a concomitant increase in the M+0 species, but this specific effect could not be easily rationalized. Entry of ^13^C from glucose through pyruvate would generate mass isotopomers with mass M+2, increasing up to M+6 with repeated cycling through the TCA cycle with no M+1 produced (Supplementary Fig. 3), which was consistent with our findings. However, anaplerotic flux through pyruvate carboxylase could also produce additional M+3 and M+5. Anaplerosis from glutamine through α-ketoglutarate would generate mass isotopologues with mass M+4, decreasing to M+0 with repeated cycling through the TCA cycle but with no M+3 produced (Supplementary Fig. 4), again consistent with our findings (Supplementary Fig. 2b). Reductive carboxylation would generate an accumulation of the M+5 mass isotopomer of citrate after incubation with [U-^13^C_5_]glutamine, which we did not observe (Supplementary Fig. 2b).

### wt- and G93ASOD1 Expression in NSC-34 Cells Stabilizes HIF-1α and Increases Expression of Glycolytic Enzymes

**Fig. 8:**
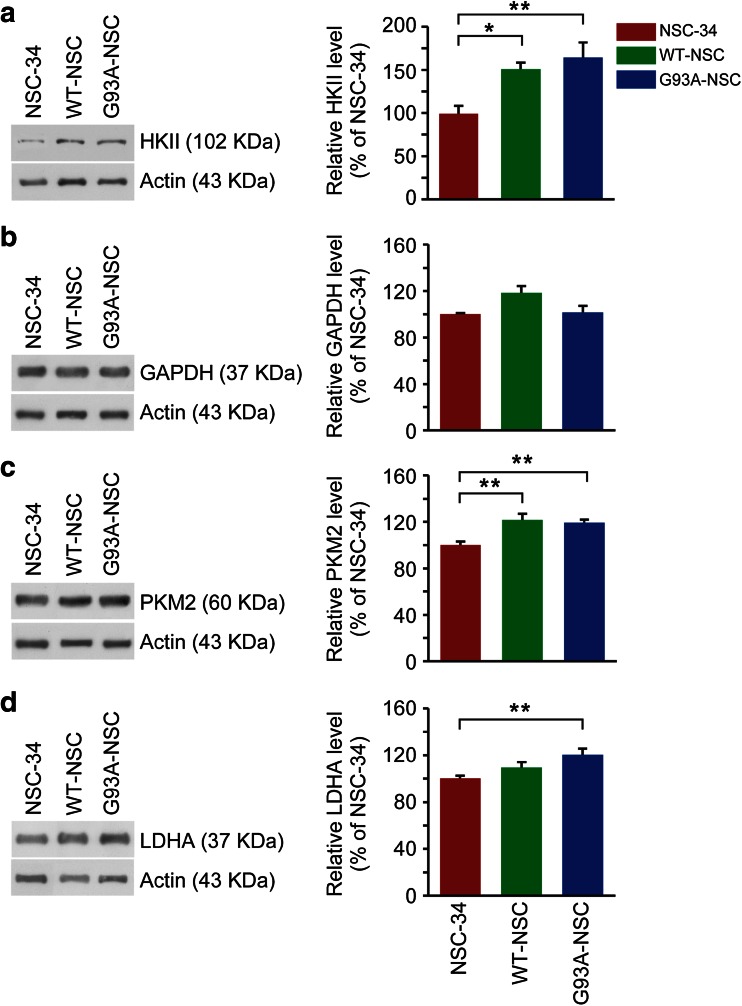
Protein expression of glycolytic enzymes of NSC-34, wtSOD1, and G93ASOD1 cells. The NSC-34, WT-NSC, and G93A-NSC cell lines were cultured for 22 h without serum. Protein expression of **a** hexokinase II (HK II; *n* = 9), **b** glyceraldehyde 3-phosphate dehydrogenase (GAPDH; *n* = 3), **c** pyruvate kinase M2 isoform (PKM2; *n* = 6), and **d** lactate dehydrogenase A (LDHA; *n* = 18) were determined by Western blot and normalized to actin. A representative Western blot is shown for each protein. All values are percentages of the NSC-34 level and are mean ± s.e.m. (*p < 0.05 and **p < 0.01 after one-way ANOVA and Tukey’s post hoc test)

Our metabolomic data provided direct evidence that wtSOD1 expression in NSC-34 cells under serum deprivation increased glycolysis to lactate, with accumulation of intracellular pyruvate, PEP, fumarate, and malate derived from glucose. Expression of G93ASOD1 recapitulated this phenotype but further increased lactate production, apparently by shifting some of the flow of pyruvate from entry into the TCA cycle towards conversion to lactate and not by regulating upstream steps in glycolysis. To validate these hypotheses, we measured the protein expression levels of the glycolytic enzymes glyceraldehyde 3-phosphate dehydrogenase (GAPDH) and the inducible forms of hexokinase 2 (HKII), pyruvate kinase M2 (PKM2), and lactate dehydrogenase A (LDHA). Levels of HKII, which regulates the first step of glycolysis, were slightly higher in the WT-NSC and G93A-NSC than in NSC-34 (+49 %, *p* < 0.05 and +63 %, *p* < 0.01, respectively, Fig. 8a), consistent with the increase in glycolytic flux in these lines. GAPDH, which catalyzes the oxidation of glyceraldehyde-3-phosphate alongside the reduction of NAD^+^, showed no significant changes (Fig. 8b). PKM2 levels were slightly but significantly higher (+20 %) in both the WT-NSC and G93A-NSC than in NSC-34 (Fig. 8c), which may contribute in part to the higher intracellular pyruvate in the WT-NSC and G93A-NSC (Fig. 4). The increase in the M2 isoform of pyruvate kinase may also indicate a more subtle regulation of pyruvate kinase activity as this isoform responds not only to the availability of its substrate but also to the upstream glycolytic intermediate fructose-1,6-biphosphate, the amino acid serine, and phosphorylation events [23, 24]. LDHA, which reduces pyruvate to lactate at the expense of NADH oxidation, was significantly higher (+20 %, *p* < 0.01) only in the G93A-NSC line compared to NSC-34 (Fig. 8d), which may contribute to the higher lactate concentration in the medium of the G93A-NSC.

**Fig. 9:**
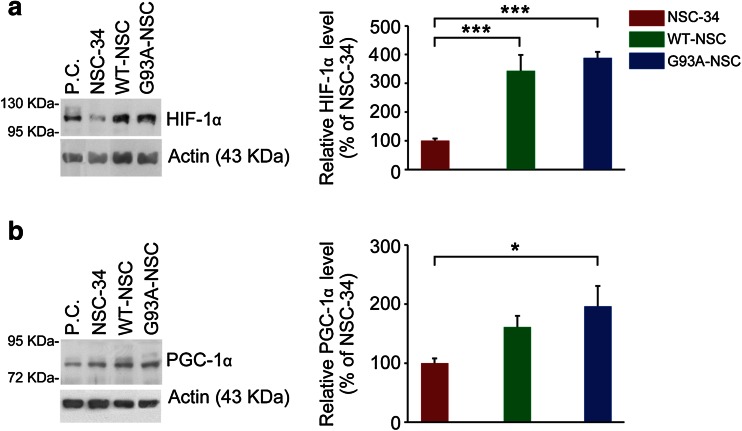
Protein expression of hypoxia-inducible factor 1 α and peroxisome proliferator-activated receptor γ co-activator-1α in the nuclei of NSC-34, wtSOD1, and G93ASOD1 cells. The NSC-34, WT-NSC, and G93A-NSC cell lines were cultured for 22 h without serum. Protein expression in nuclear lysates of **a** hypoxia-inducible factor 1 α (HIF-1α; *n* = 6) and **b** nuclear peroxisome proliferator-activated receptor γ co-activator-1α (PGC-1α; *n* = 10) were determined by Western blot and normalized to actin. A representative Western blot is shown for each protein. P.C. indicates a positive control sample, i.e., a cell lysate of NSC-34 cells treated with 1 mM dimethyloxaloylglycine (DMOG) for 4 h for HIF-1α and a cell lysate of DU145 cells for PGC-1α. Mean ± s.e.m. percentages of the NSC-34 level are shown (*p < 0.05, and ***p < 0.001 after one-way ANOVA and Tukey’s post hoc test)

The higher levels of HKII and PKM2 proteins in the WT-NSC and G93A-NSC, and of LDHA in the G93A-NSC resemble the cell-autonomous reprogramming of glucose and energy metabolism activated by HIF-1α. Expression of HIF-1α protein in the cell nuclei was significantly higher in both the WT-NSC and G93A-NSC than in NSC-34, indicating that SOD1 expression activated a pseudohypoxic state (Fig. 9a). However, there was no significant difference between the WT-NSC and G93A-NSC.

We then determined the levels of the peroxisome proliferator-activated receptor γ co-activator-1α (PGC-1α), which is involved in the promotion of mitochondrial biogenesis and respiration [25] but also regulates ROS removal under oxidative stress [26]. Nuclear PGC-1α protein tended to be higher in the WT-NSC and G93A-NSC (+61 % n.s. and +96 % *p* < 0.001, respectively, Fig. 9b) than in NSC-34. This mirrored the increases in glycolysis, suggesting that PGC-1α upregulation may be a response to enhance clearance of oxidative stress caused by serum deprivation rather than to enhance mitochondrial respiration.

### Increased Lactate Accumulation in the G93A-NSC Cell Line Is Associated with Pyruvate Dehydrogenase Kinase 1 Induction

**Fig. 10:**
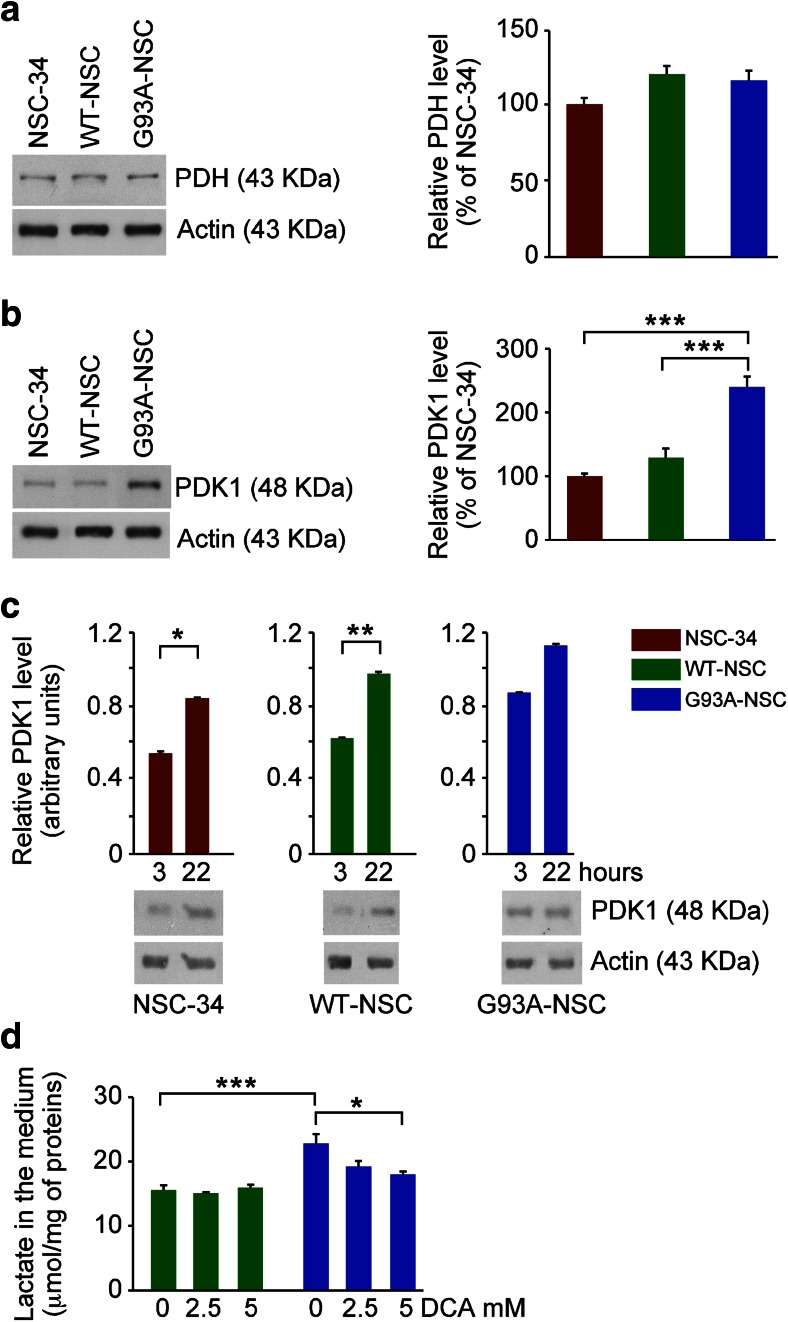
Lactate accumulation in the G93A-NSC cell line is associated with pyruvate dehydrogenase kinase 1 induction and treatment with dichloroacetate lowers the lactate level. Levels of **a** pyruvate dehydrogenase α 1 subunit (PDH; *n* = 3) and **b** pyruvate dehydrogenase kinase 1 (PDK1; *n* = 12) of NSC-34, WT-NSC, and G93A-NSC cell lines cultured for 22 h without serum were determined by Western blot and normalized to actin. Percentages of the NSC-34 level are shown and a representative Western blot for each protein (***p < 0.001 after one-way ANOVA and Tukey’s post hoc test). **c** Time-course (3 and 22 h) of PDK1 levels (normalized to actin; *n* = 3) of NSC-34, WT-NSC, and G93A-NSC cell lines cultured without serum (*p < 0.05 and **p < 0.01 by Student’s *t* test). **d** Levels of lactate (μmol/mg of proteins; *n* = 3) in the medium of WT-NSC and G93A-NSC cultured without serum for 46 h with/without the PDK1 inhibitor sodium dichloroacetate (DCA; 2.5 and 5 mM; *p < 0.05 and ***p < 0.001 after one-way ANOVA and Tukey’s post hoc test). All values are shown as mean ± s.e.m

To further investigate the cause of lactate accumulation, we investigated whether the entry of pyruvate into the TCA cycle in the G93A-NSC was limited by the restriction of pyruvate dehydrogenase complex activity, which metabolizes pyruvate to acetyl-CoA. This complex comprises three enzymes, the first being pyruvate dehydrogenase; the activity of the complex is regulated by the expression of its constituent proteins or by phosphorylation/dephosphorylation of the α subunit of pyruvate dehydrogenase (PDH) [27]. The expression of PDH protein was not significantly modified in any of the cell lines after serum deprivation (Fig. 10a). Pyruvate dehydrogenase kinase 1 (PDK1) is the main inhibitory kinase regulating PDH activity in neurons and astrocytes [28] and its induction attenuates ROS formation by the mitochondrial electron transport chain [29]. In previous studies, we found that a high level of PDK1 protein was a response to stressful conditions in NSC-34 cells expressing high/low levels of G93ASOD1 [18, 20]. Similarly, after 22 h of serum deprivation, PDK1 protein levels were significantly higher in the G93A-NSC line (Fig. 10b) than in NSC-34 (+137 %) or WT-NSC (+109 %).

We then compared PDK1 protein levels of each cell line after 3 h and after 22 h of serum deprivation (Fig. 10c). There was a significant increase (+50 %) in the NSC-34 and WT-NSC lines, but not in the G93A-NSC (+25 %). This suggests that culture without serum may favor an increase in PDK1 in all the cell lines, but this was not the main factor driving PDK1 induction in the G93A-NSC, where after 22 h, PDK1 levels were significantly higher than both NSC-34 and WT-NSC (Fig. 10b). To investigate the role of PDH and PDK1 in the lactate accumulation, we treated the WT-NSC and G93A-NSC lines with DCA, an inhibitor of PDKs that permits full activity of PDH [30]. Treatment with 5 mM DCA for 46 h significantly reduced lactate in the medium of the G93A-NSC line (Fig. 10d). Lower concentrations and shorter treatments with DCA were not sufficient to observe changes in extracellular lactate. DCA treatment did not significantly modify the viability of the cell lines (Supplementary Fig. 5). We also considered the possibility that lactate transporters were upregulated, but there were no changes in the protein expression level of the monocarboxylate transporter 1 (MCT1) across the cell lines (Supplementary Fig. 6). However, cells tightly regulate levels of intracellular lactate to maintain pH, as acidification from intracellular lactate accumulation can limit the glycolytic capacity of cells [31]. With the elevation of glycolysis from SOD1 overexpression leading to increased lactate, and the further increase from the inactivation of PDH by PDK1 in the G93A-NSC line, the cell lines may also engage in the export of the lactate that exceeds the cell’s buffering capacity and tolerable pH limits through increased monocarboxylate transporter activity. This would be consistent with the increase in extracellular lactate observed in WT-NSC and further in G93A-NSC (Figs. 1 and 2) together with the increase in intracellular lactate in both SOD1-overexpressing lines (with no difference in WT-NSC and G93A-NSC; Fig. 4b). Overall, results strongly implicate PDK1 inactivation of PDH as an upstream cause of the more marked accumulation of intracellular pyruvate and extracellular lactate in the G93A-NSC line than in WT-NSC (Figs. 1, 2, and 4b).

### Intracellular Levels of Amino Acids Are Lower in the G93A-NSC Line Than in the WT-NSC and NSC-34

During serum deprivation, promotion of cell survival involves the activation of the integrated stress response (ISR), which ensures the supply of amino acids for protein and glutathione biosynthesis [32, 33]. We therefore examined the intracellular concentrations of several amino acids in the cell lines.

**Fig. 11:**
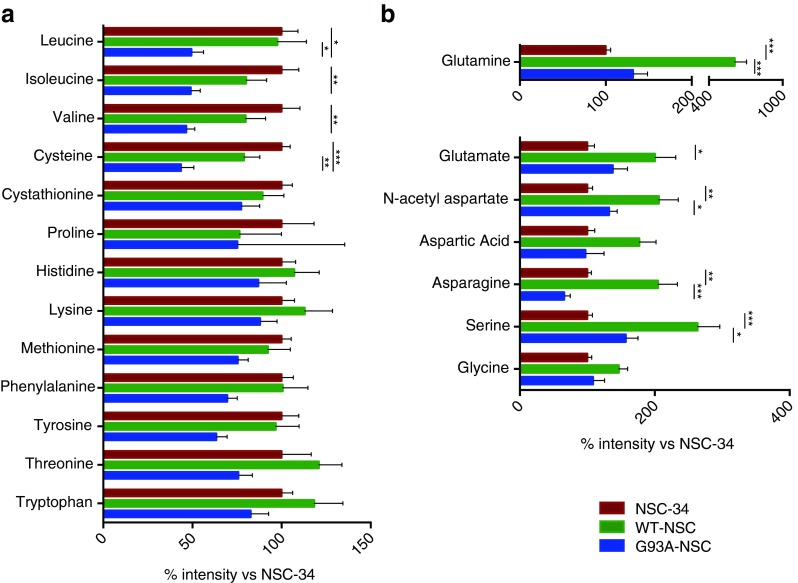
Modulation of intracellular amino acid pools in the WT-NSC and G93A-NSC cell lines. The intracellular levels of amino acids of NSC-34, WT-NSC, and G93A-NSC cell lines cultured for 22 h without serum were measured by GC-MS and expressed as percentages of the levels in the untransfected NSC-34 line. All values are mean ± s.e.m. (*n* = 3 for proline; *n* = 6 for all other amino acids. *p < 0.05, **p < 0.01, ***p < 0.001 after one-way ANOVA with FDR correction for multiple testing and Tukey’s post hoc test)

Broadly, the changes in amino acid levels (Fig. 11) could be divided into two groups. The first group showed no significant difference with wtSOD1 expression compared to the untransfected NSC-34, but had lower levels specifically with G93ASOD1 expression. This group was dominated by essential/semi-essential amino acids including the branched-chain amino acids leucine, isoleucine, and valine (significant decreases in G93A-NSC of 51, 51, and 53 % vs. NSC-34 respectively, *p* < 0.05) and cysteine (−56 % compared to NSC-34, *p* < 0.001), as well as proline, histidine, lysine, methionine, phenylalanine, threonine, tryptophan, and tyrosine (non-significant decreases ranging 10–55 %, Fig. 11a). In contrast, the second group of amino acids exhibited large increases with wtSOD1 expression compared to NSC-34. These amino acids can be synthesized de novo and are involved in neurotransmission, as they are precursors in the synthesis or act directly as neurotransmitters (Fig. 11b). We observed significantly higher levels of serine (+164 %), N-acetyl aspartate (+106 %), asparagine (+105 %), glutamine (+511 %), and glutamate (+100 %) and non-significant increases in glycine (+47 %) and aspartate (+77 %). However, this effect was absent or less pronounced in the G93A-NSC line (−40 %, −36 %, −78 %, −68 % in the G93A-NSC compared to WT-NSC for serine, N-acetyl aspartate, glutamine, and asparagine, respectively, *p* < 0.05), with no significant increases compared to NSC-34. These results indicate a widespread decrease in amino acid availability in the G93ASOD1-expressing cells under serum deprivation compared to the wtSOD1.

**Fig. 12:**
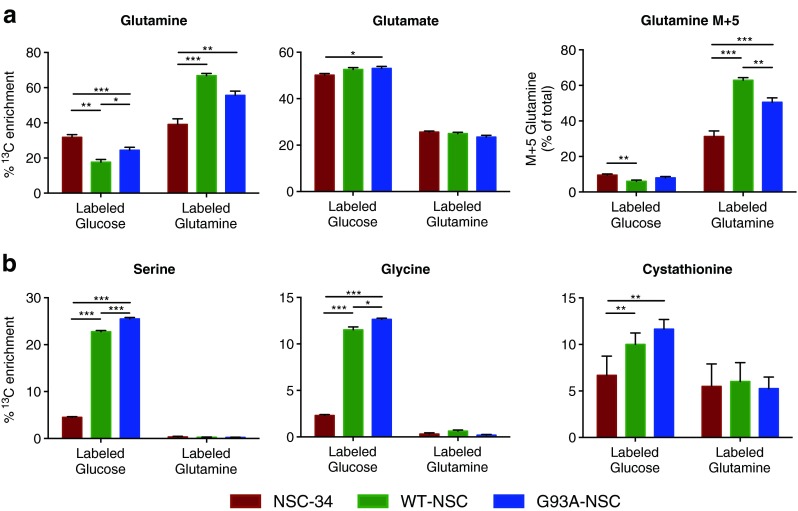
Glutamine, glutamate, glycine, serine, and cystathionine are differently enriched with ^13^C in the G93A-NSC line. The NSC-34, WT-NSC, and G93A-NSC cell lines were cultured for 22 h without serum and with [U-^13^C_6_]glucose or [U-^13^C_5_]glutamine. Histograms show the percentages (mean ± s.e.m.) of the intracellular pools of **a** glutamine and glutamate, and **b** the glutathione precursors glycine, serine, and cystathionine enriched with ^13^C after incubation with labeled glucose or labeled glutamine (*n* = 5, *p < 0.05, **p < 0.01, ***p < 0.001 after one-way ANOVA with FDR correction for multiple testing and Tukey’s post hoc test)

Investigation of the ^13^C enrichment of amino acid pools through culture with labeled glucose and glutamine confirmed that for five amino acids there was significant de novo synthesis: these were glutamine, glutamate, serine, glycine, and cystathionine (precursor of cysteine). After incubation with labeled glucose at least 50 % of the intracellular glutamate was enriched with ^13^C, with a small but significant increase for G93A-NSC compared to NSC-34 (+0.9 %, *p* < 0.05). Interestingly, a smaller proportion of intracellular glutamate (20–25 %) was derived from glutamine, which is the canonical pathway for glutamate synthesis in neurons in vivo. There was substantial ^13^C enrichment from [U-^13^C_6_]glucose of intracellular glutamine, and levels differed significantly between the lines (32 % in NSC-34; a decrease to 18 % in WT-NSC and 24 % in G93A-NSC, *p* < 0.05; Fig. 12a). When incubated with [U-^13^C_5_]glutamine, the pattern of ^13^C enrichment in intracellular glutamine was reversed (39 % in NSC-34, 67 % in WT-NSC, and 56 % in G93A-NSC). The presence of the M+5 isotopologue of glutamine in cells grown on [U-^13^C_6_]glucose (Fig. 12a) indicated the activity of glutamine synthetase (GS). However differences in the overall ^13^C enrichment of glutamine among the three genotypes could not be attributed to different protein expression levels of GS (Supplementary Fig. 7), suggesting that wtSOD1 increases glutamine transport into the cell and/or affects glutaminase activity. Surprisingly, the differences had no effect on overall ^13^C enrichment of the glutamate pool (Fig. 12a).

Besides being involved in the synthesis of TCA cycle metabolites (Fig. 5) or in glutamine metabolism (Fig. 12a), glutamate plays a role in other metabolic pathways [34] including the synthesis of other amino acids (serine and glycine) and of the antioxidant glutathione. We examined the level of ^13^C enrichment from labeled glucose or glutamine of the intracellular pools of serine and glycine, which are formed sequentially, and of cystathionine, the precursor of cysteine (Fig. 12b). Glycine and cysteine, with glutamate, are also precursors of glutathione. Production from glucose contributed to a larger percentage of glutamate, glycine, and cystathionine than glutamine. There was also a marked and significant increase in ^13^C enrichment of serine and glycine from labeled glucose in both the WT-NSC and G93A-NSC, compared to NSC-34, with significantly more labeling from glucose in G93A-NSC than in WT-NSC (Fig. 12b), confirming the activation of serine synthesis by serum deprivation in the SOD1-transfected lines (Fig. 4a). ^13^C enrichment of cystathionine was also significantly increased in both WT-NSC and G93A-NSC (more marked in the latter) suggesting that glutathione synthesis might be increased by serum deprivation in these lines, more than in NSC-34. Activation of serine and glutathione synthesis in the WT-NSC and G93A-NSC may help explain why ^13^C enrichment of the glutamate pool in these lines was comparable to that of the NSC-34 (Fig. 12a).

## Discussion

Clinical and experimental evidence indicate that defects in energy metabolism may be pathogenic in ALS [7, 8]. The dysfunction of motor neuron mitochondria typical of this disease [4, 5] may play a contributory role, as neurons rely on mitochondrial oxidative phosphorylation as their main source of energy [30]. Bioenergetic defects have previously been described in motor neuronal cells expressing different mutant forms of SOD1 including G93ASOD1, as well as in fibroblasts isolated from ALS cases, expressing I113T SOD1, and the adaptation of energy metabolism in response to stimuli was associated with toxicity [6, 20, 35].

The present study employed a well-characterized motor neuronal ALS model expressing the mutant G93ASOD1 in the NSC-34 cell line, a spinal cord neuron-neuroblastoma hybrid, which has become the most widely used line to study mechanisms of motor neuron degeneration. The degree of functional relevance of results in proliferating cell lines in comparison to those in non-dividing cells may vary; however, the cell lines are suited for high-throughput analysis as they can be propagated without limitation. In studies such as ours, a particular concern will be the impact of metabolic alterations related to oncogenic transformation on the results of a broad metabolomic analysis. We have focused on changes relating to the expression of mutant human SOD1 in comparison with both the expression of wild-type human SOD1 and the untransfected line, in order to limit the impact of the neuroblastoma phenotype on interpretation. In addition, evidence from a recent deep proteomic comparison of primary motor neurons and NSC-34 cells indicates that metabolic and housekeeping pathways are conserved as regards the proteins involved and their abundance [36], suggesting that the NSC-34 line may continue to be useful for such metabolic investigations.

We generated a detailed characterization of metabolism through untargeted metabolite profiling and stable isotope-incorporation analysis using ^1^HN MR spectroscopy and GC-MS. This approach enabled us to assess the effect of mutant SOD1 on interacting metabolic pathways supported by glucose and glutamine and provided measurements of novel metabolic changes. The results suggest links between different alterations such as mitochondrial dysfunction, oxidative stress, and impairment of the endoplasmic reticulum (ER) stress response, which are associated with neurodegeneration in ALS patients [37, 4] and are seen in animal and cellular models of ALS-expressing mutant forms of SOD1 [38].

The initial observation was that under serum deprivation, glycolytic protein expression, and glycolytic flux to lactate were increased in both WT-NSC and G93A-NSC compared to the untransfected line. This appears consistent with the proposed role of SOD1 to repress glucose-mediated respiration [3]. We and other groups have previously shown that even under basal conditions, G93ASOD1 and other mutant SOD1s increase the formation of ROS and reactive nitrogen species and alter mitochondrial morphology in the NSC-34 line [13, 14, 39]. Serum deprivation also substantially increases the formation of ROS and reactive nitrogen species [14, 40], which are known inhibitors of the mitochondrial electron transport chain complexes [41]. This increase in ROS formation may trigger SOD1 regulation of respiration and hence glycolysis, as SOD1-mediated signaling occurs through stabilization of casein kinase 1-γ homologs by catalyzing superoxide conversion to peroxide [3]. HIF-1α stabilization was also more marked in both the WT-NSC and G93A-NSC than in the untransfected line, suggesting the development of a pseudo-hypoxic state. However, there was no clear difference in HIF-1α levels between WT-NSC and G93A-NSC, indicating that pseudohypoxia was being caused by SOD1 overexpression, but was not leading to the excess lactate production in the G93A-NSC. Under normoxic conditions, high hydrogen peroxide levels can act as a hypoxia mimetic, promoting HIF-1α stabilization [42]. In the WT-NSC and G93A-NSC, the accumulation of pyruvate and fumarate may also contribute to the HIF-1α increase as these metabolites can compete with the substrate α-ketoglutarate in the hydroxylation reaction by which the HIF prolyl-hydroxylases target HIF-1α for degradation [43, 22].

Comparison of WT-NSC and G93A-NSC showed that the specific loss of viability of the G93A-NSC line was accompanied by a significantly higher net flux to lactate in the G93A-NSC, suggesting that this metabolic change may influence survival. To explain this change in the G93A-NSC, we showed that the protein expression levels of PDK1 and LDHA (which are critical for lactate overproduction) increased while the glucose-derived acetyl-CoA flux into citrate decreased. PDK1 induction is a HIF-1-mediated response to mitigate ROS formation by mitochondria primarily utilizing glucose as a substrate for oxidative phosphorylation [44] by repressing mitochondrial function and oxygen consumption as a consequence of the shift of pyruvate to lactate. In the G93A-NSC, the induction of PDK1 and LDHA may be driven by the need to divert pyruvate derived from glycolysis away from oxidative metabolism and reduce the burden of mitochondrial free radical formation. Our results in the G93A-NSC line agree with findings in fibroblasts from ALS cases carrying I113T *SOD1* showing an increase in glycolytic flux and a reduction in mitochondrial respiration compared with fibroblasts from healthy controls [35].

Overexpression of PDK1 and LDHA have been shown to protect against neuronal death by reducing mitochondrial respiration and ROS production and maintaining ATP levels in a model of Alzheimer’s disease characterized by mitochondrial dysfunction [45]. In the G93A-NSC cells, the increased glycolytic flux may be used to maintain energy generation outside of mitochondrial respiration by taking advantage of the high levels of glucose present in the medium. It must be noted however, that while 25 mM glucose is used routinely in the culture of NSC-34 and other primary and immortalized neuronal culture, this is not entirely reflective of in vivo conditions. Further in vivo investigation into this adaptation will provide a clearer picture into the extent by which it may be observed in neuronal cells and impact the disease process.

Additional evidence of the adaptations to oxidative stress in the G93A-NSC line comes from the increase of PGC-1α, which can upregulate the expression of ROS-detoxifying enzymes to protect neural cells from cytotoxicity due to oxidative insult [26]. In our model, the increase in PGC-1α was parallel to that of lactate, making it unlikely that it was related to the stimulation of respiration [25]. However, despite these metabolic adaptations to mitigate mitochondrial ROS production, the increased glycolytic flux to lactate induced by wt/G93ASOD1 could paradoxically make the motor neuronal cells more vulnerable to the effects of oxidative stress by reducing pentose phosphate pathway (PPP) flux. In cortical neurons, the capacity to metabolize glucose through glycolysis was reported to be low, with glucose being used extensively in the PPP to build up reducing power (NADPH). The NADP H, in turn, is used to replenish the pool of reduced glutathione, the major cell antioxidant, and activation of neuronal glycolysis (via Pfkfb3) led to increased oxidative stress and apoptosis [46]. An increased flux of glucose through glycolysis may then limit that through PPP, increasing susceptibility to oxidative stress.

This possibility is consistent with the observation that systemic administration of DCA, the PDK1 inhibitor used in the present study, increased survival of the G93ASOD1 mouse [47]. This effect was apparently due to modification of the mitochondrial function of astrocytes, reducing astrocyte reactivity and preventing motor neuron loss. PDH phosphorylation in the astrocytes was decreased by DCA treatment, suggesting that modulation of PDK1 induction may hold potential as a therapeutic strategy for future investigation.

The WT-NSC and G93A-NSC lines also had increased biosynthesis from glucose of glutamine (which leads to glutamate), glycine, and serine and cystathionine (both leading to cysteine) than the untransfected line. This suggests that overexpression of SOD1 might support the glutathione synthesis necessary to counteract serum deprivation-induced oxidative stress by driving de novo synthesis of amino acid precursors of this cellular antioxidant. However, when considering the total pool of each amino acid precursor of glutathione, we only observed an increase in the WT-NSC line compared to the NSC-34; the G93A-NSC also had a smaller pool of cystathionine. The size of these pools may be a previously unrecognized limiting factor contributing to trigger oxidative stress, as a decrease in glutathione was reported in cell models with prolonged exposure/high level of mutant G93ASOD1 [48] and during disease progression in the G93ASOD1 mouse [49]. The increased serine in the WT-NSC and G93A-NSC lines is compatible with that of 3-phosphoglycerate in these lines, and indicates the induction of serine synthesis. This effect has previously been observed at disease onset in motor neurons of G37RSOD1 mice [50] and is regulated by a sensor of non-essential amino acid starvation through the GCN2-ATF4 pathway [23]. l-Serine also has a versatile role in intermediary metabolism, particularly in neuronal tissue, as it is a precursor of d-serine and glycine, both NMDA receptor co-agonists. In the G93ASOD1 mouse spinal cord, d-serine levels were progressively elevated [51]. Alterations to d-serine homeostasis have been implicated in the development of ALS, since some cases of fALS have a mutation in d-amino acid oxidase, which impairs the activity of this enzyme [52]. However, although we saw high levels of serine, we could not resolve chiral species with the metabolomic method applied. More precise measurements for each chiral species would be required to identify the specific contributions of the d- and l- stereoisomers.

Regulation of lactate levels is also an important factor in supporting neuronal survival. Disruption of lactate transport in experimental models leads to axon damage and neuron loss, and MCT1 levels were low in ALS patients [11]. We did not see any reduction of this transporter in the G93A-NSC line under serum deprivation, but the shift towards increased lactate production and release may affect the glutamine-glutamate cycle and the prevention of excitotoxicity. In astrocytes, glutamate uptake is coupled with a shift in glycolysis towards lactate production [53] while in neurons, increasing lactate uptake through increased monocarboxylate transporter 2 expression can prevent glutamate-mediated excitotoxicity [54]. In the motor neuronal G93A-NSC line, as it presented a significant increase in net lactate output, the preference was for clearance of lactate rather than utilization.

The NSC-34 cell line has very low sensitivity to glutamate [55] so it is unlikely that excitotoxicity played a role in cell death in our model. However, increased net lactate output in an astrocyte-neuron metabolic interaction in vivo may compromise glutamate uptake by astrocytes, limiting its synaptic clearance. This, coupled with the greater glutamate release in the G93A-NSC compared to WT-NSC, suggests that the metabolic changes due to mutant SOD1 expression may potentially increase motor neuron susceptibility to glutamate-mediated excitotoxicity.

The other main metabolic change was a general and significant depletion of amino acid pools in the G93A-NSC. Serum deprivation has been reported to induce ER stress, which activates the PERK/eIF2α/ATF4-dependent ISR [32] in an attempt to balance the accumulation of misfolded proteins in the ER. The PERK pathway is one of three that make up the unfolded protein response (UPR), and promotes amino acid sufficiency for the synthesis of protective proteins, and in fact, amino acid pools were larger in the WT-NSC than in G93A-NSC. ER stress, UPR, and activation of ISR are present in models expressing mutant SOD1 and in ALS patients [56, 1]. The exact mechanism through which mutant SOD1 causes ER stress is not known, although several studies suggest that misfolded mutant SOD1 is present in the ER lumen [56]. In our model, it is hard to see whether there are misfolded proteins and/or aggregates because of the low expression level of the mutant protein [13]. However, our results suggest that dysregulation of glucose metabolism by G93ASOD1 potentially alters the cell’s ability to maintain the UPR. A role for the PERK pathway has been identified in the pathogenesis of mutant SOD1-linked fALS and holds potential as a target for treatment [57]. The metabolic phenotype we observed in the G93A-NSC line would be compatible with down regulation of proteins involved in amino acid import and metabolism, as shown in Perk^−/−^ cells [33] which have impaired ISR.

The amino acid depletion in the G93A-NSC line also suggests impaired autophagy (macroautophagy), which mutant SOD1 has also been noted to affect [1]. Cells and mice deficient in autophagic genes were unable to maintain the cellular amino acid pool [58]. Activation of autophagy is a central component of the ISR and is a response to nutrient deprivation and to the failure of the ubiquitin-proteasomal system when it is overwhelmed by misfolded proteins from the ER [59]. In addition, the activation of selective mitochondrial autophagy is also a strategy to reduce ROS generation [44]. In our model, leucine, glutamine, and glutamate were more abundant in the cells expressing wtSOD1 compared to the cells expressing G93ASOD1. This suggests the possibility of dysregulation of mTORC1-mediated autophagy activation, which is controlled by leucine and glutaminolysis [60].

## Conclusions

We captured metabolomic changes in motor neuronal cells expressing wt- and mutant G93ASOD1 exposed to oxidative stress and ISR activation, as well as selective cell death in cells expressing the ALS-causing mutation. wtSOD1 orchestrated a metabolic rearrangement based on increased glucose metabolism which coordinated promotion of resistance to oxidative stress and amino acid depletion/ER stress. In the presence of the mutant SOD1, this program failed and turned into cytotoxicity. Our results provide fresh insights into the mechanisms by which defective regulation of metabolism by G93ASOD1 could promote motor neuron death and disease progression in ALS.

## Electronic Supplementary Material

Below is the link to the electronic supplementary material.

ESM 1 (PDF 681 kb)

## Abbreviations

ALS: Amyotrophic lateral sclerosis
DCA: Dichloroacetate
ER: Endoplasmic reticulum
fALS: Familial amyotrophic lateral sclerosis
FDR: False-discovery rate
G93ASOD1: Human mutant G93A Cu/Zn superoxide dismutase
GAPDH: Glyceraldehyde 3-phosphate dehydrogenase
GC-MS: Gas chromatography–mass spectrometry
GS: Glutamine synthetase
HIF-1α: Hypoxia inducible factor 1α
HKII: Hexokinase II
ISR: Integrated stress response
LDHA: Lactate dehydrogenase A
MCT1: Monocarboxylate transporter 1
^1^H NMR: 1H nuclear magnetic resonance
PDH: Pyruvate dehydrogenase α subunit
PDK1: Pyruvate dehydrogenase kinase 1
PEP: Phosphoenolpyruvate
PGC-1α: Peroxisome proliferator-activated receptor γ co-activator-1α
PKM2: Pyruvate kinase M2
PPP: Pentose phosphate pathway
ROS: Reactive oxygen species
sALS: Sporadic amyotrophic lateral sclerosis
SOD1: Cu/Zn superoxide dismutase
TCA: Tricarboxylic acid
UPR: Unfolded protein response
wtSOD1: Human wild-type Cu/Zn superoxide dismutase.
