# Supplementary material for: Metabolomic Analysis Reveals Increased Aerobic Glycolysis and Amino Acid Deficit in a Cellular Model of Amyotrophic Lateral Sclerosis
Source: Mol Neurobiol. 2015 May 12;53:2222–40. doi: 10.1007/s12035-015-9165-7 (PMC4823370; doi:10.1007/s12035-015-9165-7)
Supplement: Supplementary file 1 — (PDF 681 kb) [file 12035_2015_9165_MOESM1_ESM.pdf]

SUPPLEMENTARY FIGURES

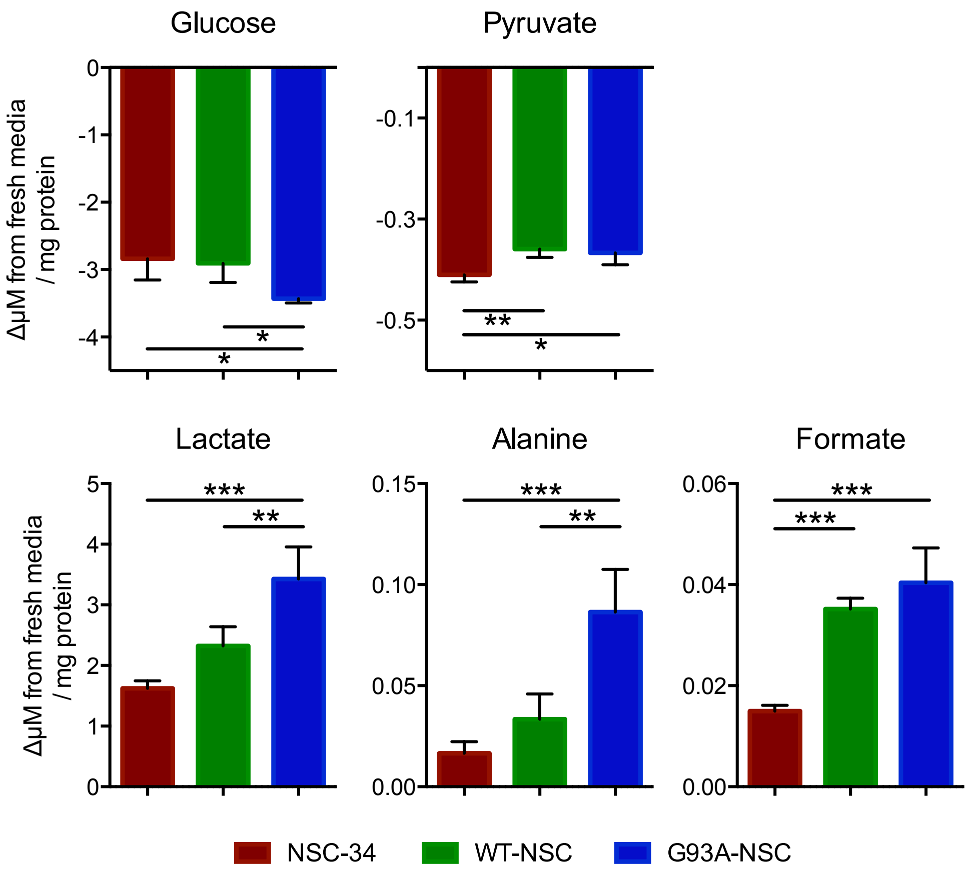

Supplementary Figure 1. **Extracellular metabolome characterization reveals regulation of glycolysis with expression of wt- and G93ASOD1 under culture with 5% FBS.** The NSC-34, WT-NSC and G93A-NSC cell lines were cultured for 22 h with 5% FBS, the media was collected and metabolites were determined by ^{1}H NMR spectroscopy. All values are mean ± s.e.m. (n=4,* p<0.05, ** p<0.01, *** p<0.001 after one-way ANOVA with False-Discovery Rate (FDR) correction for multiple testing and Tukey's post-hoc test).

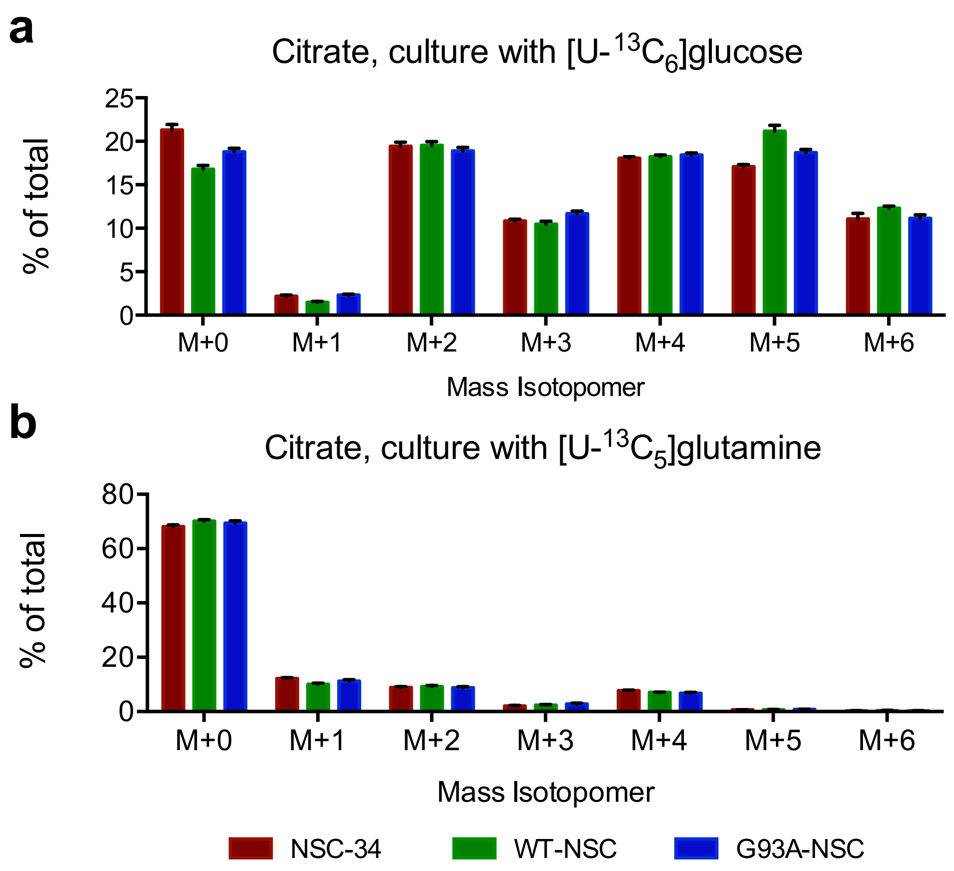

Supplementary Figure 2. **Effect of wt- and G93ASOD1 expression on the mass isotopomer distribution of citrate after culture with labeled glucose or glutamine.** The NSC-34, WT-NSC and G93A-NSC cell lines were cultured for 22 h without serum and with [U-$^{13}C_6$]glucose or [U-$^{13}C_5$]glutamine. Histograms show the mass isotopomer distribution of citrate after culture with (a) labeled glucose or (b) labeled glutamine.All values are mean ± s.e.m. (n=5).

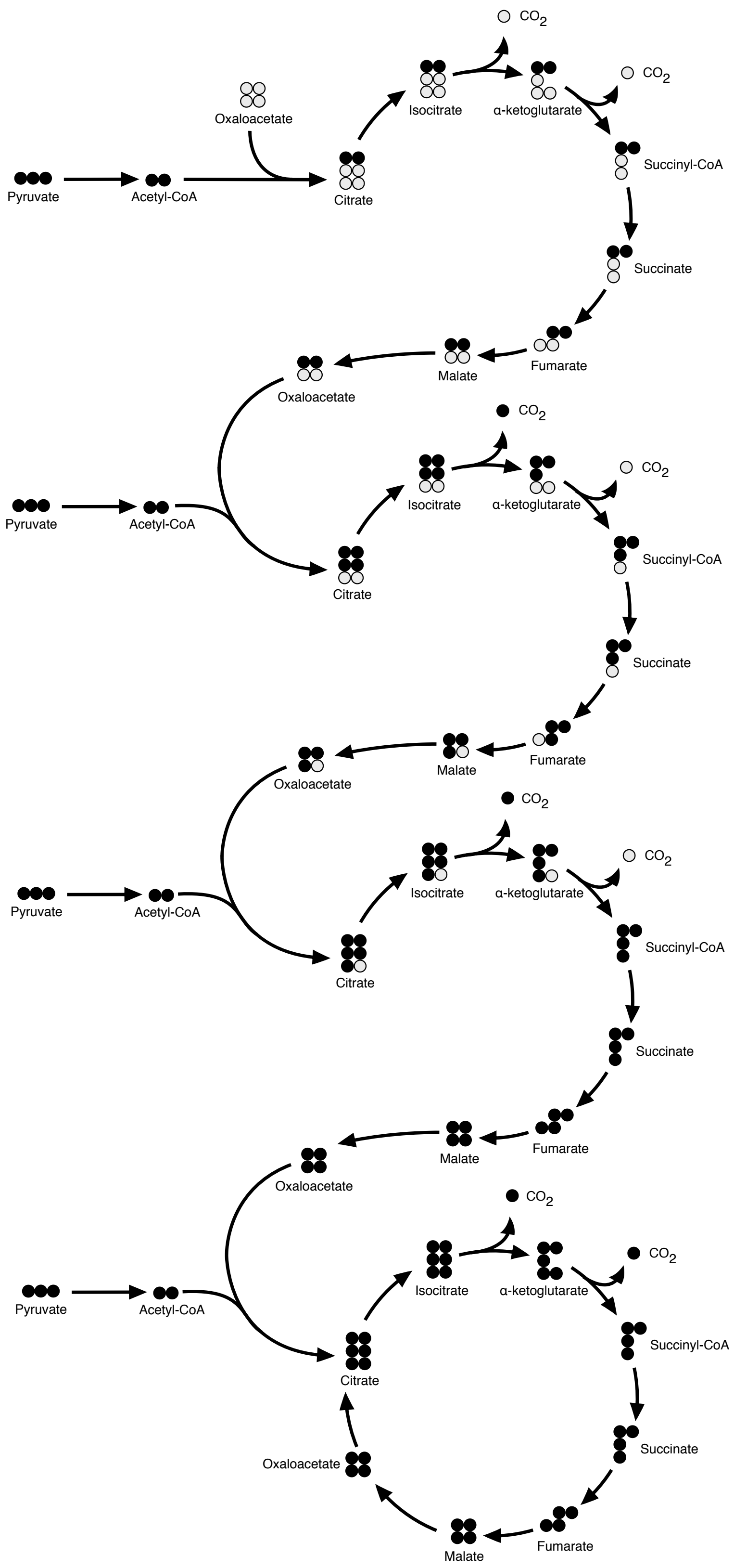

Supplementary Figure 3. **Labeling scheme from U-^{13}C pyruvate entry into the TCA cycle after culture with [U-$^{13}C_6$]glucose.** Potential mass isotopomers for TCA cycle intermediates after entry of [U-$^{13}C_3$]pyruvate from [U-$^{13}C_6$]glucose in repeated cycling through the TCA cycle.

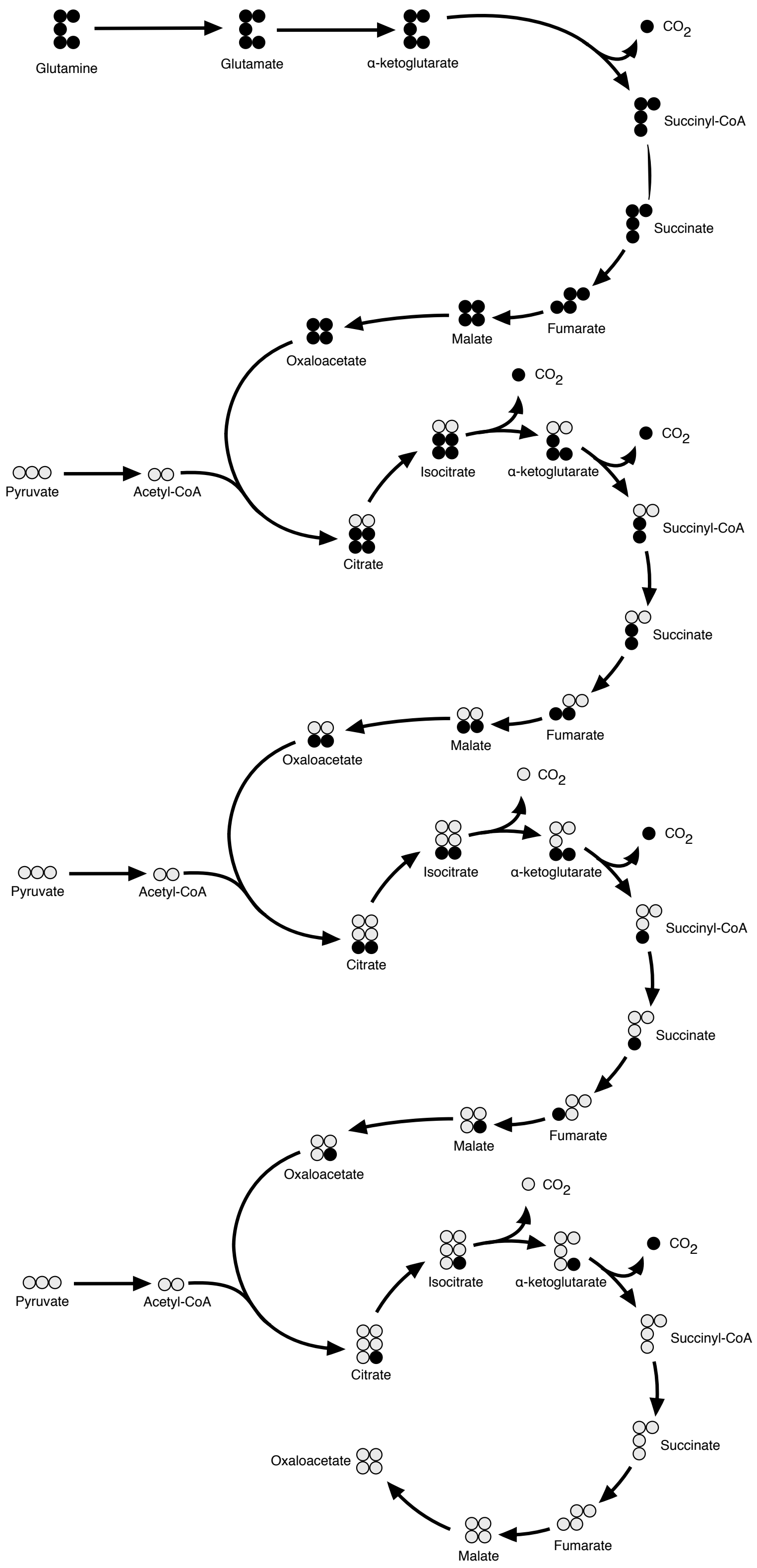

Supplementary Figure 4. **Labeling scheme of ^{13}C incorporation into the TCA cycle after culture with [U-$^{13}C_5$]glutamine.** Potential mass isotopomers for TCA cycle intermediates after labeling with [U-$^{13}C_5$]glutamine and subsequent dilution of ^{13}C with the entry of ^{12}C pyruvate in repeated cycles.

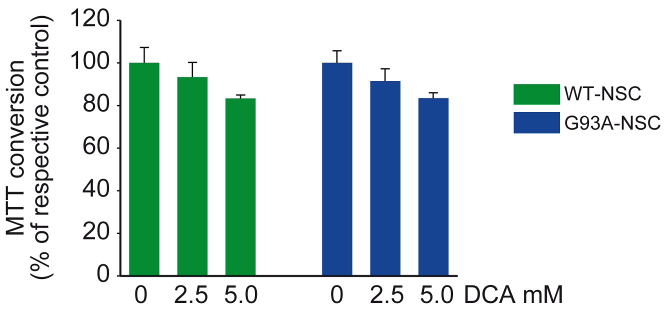

Supplementary Figure 5. **Viability of the WT-NSC and G93A-NSC cell lines after treatment with sodium dichloroacetate.** The WT-NSC and the G93A-NSC cell lines were cultured without serum for 46 h with/without sodium dichloroacetate (DCA) 2.5mM and 5mM. Viability of the cell lines was determined with the MTT assay. Values (mean ± s.e.m.) are percentages of the MTT conversion after culture without DCA (100%) (n = 3).

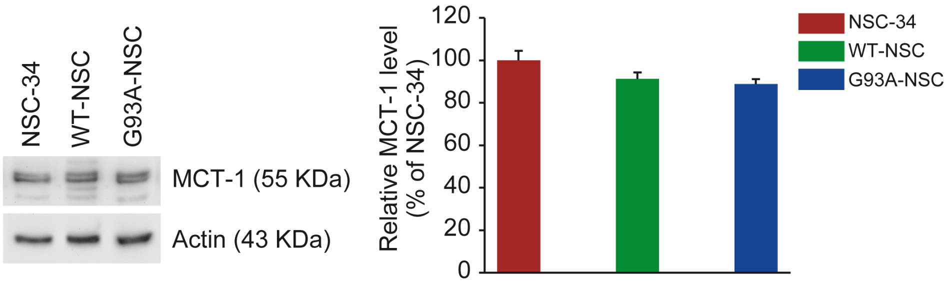

Supplementary Figure 6. **Expression level of the monocarboxylate transporter 1 protein of the NSC-34, WT-NSC and G93A-NSC cell lines.** The NSC-34, WT-NSC and G93A-NSC cell lines were cultured without serum for 22 h and the levels of monocarboxylate transporter 1 (MCT1) protein were determined by Western blot and normalized to actin. The value of the NSC-34 cell line was taken as 100%. Means ± s.e.m. (n=6) and a representative Western blot are shown.

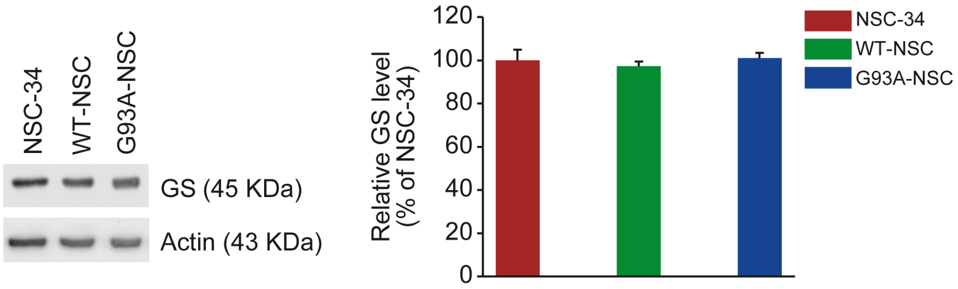

Supplementary Figure 7. **Expression level of glutamine synthetase protein of the NSC-34, WT-NSC and G93A-NSC cell lines.** The NSC-34, WT-NSC and G93A-NSC cell lines were cultured without serum for 22 h and the levels of glutamine synthetase (GS) protein were determined by Western blot and normalized to actin. The value of the NSC-34 cell line was taken as 100%. Values are shown as mean ± s.e.m. (n=3) and a representative Western blot is presented.
